# Rhythmia zero-fluoroscopy workflow with high-power, short-duration ablation: retrospective analysis of procedural data

**DOI:** 10.1007/s10840-022-01283-0

**Published:** 2022-06-28

**Authors:** Jose R. Cuellar-Silva, Elizabeth M. Albrecht, Brad S. Sutton

**Affiliations:** 1 Cardiac Rhythm Center Houston, 250 Blossom Street Suite 275, Webster, TX USA; 2 Boston Scientific Corp., St. Paul, MN USA

**Keywords:** Atrial fibrillation, Catheter ablation, Zero fluoroscopy, High-density mapping

## Abstract

**Background:**

Fluoroscopy is commonly used during atrial fibrillation (AF) ablation to guide catheter navigation and placement. Technology improvements have significantly reduced fluoroscopy time, and subsequent radiation dose, necessary to perform successful ablations. However, there is still no amount of radiation exposure known to be completely safe. The aim of this manuscript is to describe a detailed zero-fluoroscopy RHYTHMIA HDx workflow for AF ablation.

**Methods:**

This was an observational, single-center experience to describe the technique, acute procedural success, and safety using a novel zero-fluoroscopy workflow with the RHYTHMIA HDx mapping system and intracardiac echocardiography (ICE). Seventy-two consecutive patients undergoing de novo or redo AF ablation were retrospectively analyzed. Venous access was guided with ultrasound. ICE combined with the mapping system’s magnetic tracking and sheath detection was used for precise catheter placement in the coronary sinus, at the transseptal puncture, and in the left atrium. A high-power, short-duration ablation strategy guided by local impedance was used. Pulmonary vein isolation was performed or touched up for all patients with additional lines added at the operator’s discretion.

**Results:**

Using this zero-fluoroscopy workflow, all patients achieved acute isolation with no significant procedure-related complications. Average procedure time was 73.7 ± 16.2 min, which included persistent (58%) and paroxysmal (42%) AF cases, and no procedures required conversion to fluoroscopy.

**Conclusions:**

In this experience, a zero-fluoroscopy workflow using the RHYTHMIA HDx mapping system combined with ICE was feasible and safe for ablation in a heterogenous AF population. This approach, in the appropriate patient population, can eliminate radiation exposure to patients and staff.

## Introduction

Atrial fibrillation (AF) is the most common arrhythmia, with an estimated prevalence of 2–4% of the adult population worldwide [1]. AF prevalence is expected to continue growing, in part due to the aging population, increased prevalence of risk factors such as obesity and hypertension, and improvements in AF detection [2, 3]. Coupled with clinical evidence continuing to support catheter ablation as an effective and safe rhythm-control strategy [4, 5], the number of AF ablation procedures performed annually has significantly risen [6–8]. With this increase in ablation procedures, it is important to find ways to reduce unnecessary radiation exposure for both patients and medical staff.

Fluoroscopy is commonly used during AF ablation procedures to guide catheter navigation and placement—often required to perform the transseptal puncture and appropriately visualize left atrial (LA) anatomy [9]. Technology improvements over the last decade, including the integration of contact force, 3-dimensional (3D) electroanatomical mapping (EAM), and intracardiac echocardiography (ICE), have significantly reduced the amount of fluoroscopy time, and subsequent radiation dose, necessary to perform a successful ablation procedure [10]. However, there is still no amount of radiation exposure that is known to be completely safe, and, therefore, it is critical to continue to reduce radiation exposure to levels as low as reasonably achievable (ALARA principle).

Significant efforts have been made within the electrophysiology community to adapt techniques and workflows to minimize the use of fluoroscopy. Several studies have demonstrated successful implementation of a zero- or near zero-fluoroscopy AF ablation workflow using the CARTO (Biosense Webster, Inc., Diamond Bar, CA, USA) [11] and Ensite NavX (Abbott, Inc., Minneapolis, MN, USA) [12–15] mapping systems while achieving reasonable procedure durations and comparable acute safety and efficacy compared to conventional, fluoroscopy-guided procedures. However, a zero-fluoroscopy left-sided workflow using the RHYTHMIA HDx (RHYTHMIA) mapping system (Boston Scientific, Marlborough, MA, USA) to perform AF ablation has not yet been described. The aim of this manuscript is to describe a detailed workflow for zero-fluoroscopy in a heterogeneous AF patient population in a single-center experience.

## Methods

This was an observational, single-center experience to describe a novel zero-fluoroscopy workflow with the RHYTHMIA mapping system. This experience was a retrospective evaluation of data generated during standard practice; data were collected according to Institutional Guidelines. Data from 72 consecutive patients undergoing AF ablation using the novel zero fluoroscopy workflow were retrospectively analyzed. All patients had previously failed medical therapy or were intolerant to anti-arrhythmic drugs. Data from patients with implanted cardiac devices was not included in analysis. These ablation procedures consisted of de novo and redo cases in paroxysmal and persistent AF patient populations.

### Patient preparation

Prior to the procedure, patients were on uninterrupted direct oral anticoagulation except on the day of the procedure. Antiarrhythmic medications were stopped at least 5 days prior to the procedure. Patients had a transesophageal echocardiogram (TEE) prior to procedure if they were diagnosed with persistent AF or if they had skipped oral anticoagulants. No pre-procedural CT scans were performed. All procedures were performed under general anesthesia with an arterial radial line for blood pressure monitoring. Esophageal monitoring was performed in all patients using the Circa S-CATH temperature probe (CIRCA Scientific Inc).

### Devices

For all AF ablation procedures, navigation and mapping were performed using the RHYTHMIA HDx mapping system (Software 3.0 and 4.5; Boston Scientific) and the INTELLAMAP ORION mapping catheter (Boston Scientific) along with the ACUSON AcuNav ultrasound catheter (Biosense Webster). SureFlex® Steerable Guiding Sheath (Baylis Medical) or Agilis™ NxT Steerable Introducer (St. Jude Medical) were used during the procedures. A POLARIS™ Decapolar (Boston Scientific) or IBI Inquiry™ Decapolar (Abbott) catheter was placed in the coronary sinus. Transseptal puncture was performed using the NRG Transseptal Needle (Baylis Medical Company, Inc., Montreal, QC, Canada) or the BRK-1 Transseptal Needle (Abbott). Radiofrequency (RF) ablation was performed using the INTELLANAV MIFI OI ablation catheter (Boston Scientific) and the DIRECTSENSE™ Technology to measure local impedance and guide lesion formation [16, 17]. In the USA, the use of the INTELLAMAP ORION mapping catheter and the INTELLANAV MIFI OI ablation catheter for the treatment of persistent atrial fibrillation ablation is outside of the labeled indication as safety and effectiveness have not been established.

### Ablation

For all patients, a high-power, short-duration (HPSD) RF ablation strategy was used. Lesions were created using 50 W power for 7–10 s. The targeted maximum interlesion distance was 5 mm. For ablation tags, Autotag parameters were set to a stability criterion of 3 mm for 5 s of ablation. Ablation tags were color coded based on the local impedance drop during a given lesion (< 14 Ω white; 14–17 Ω pink; ≥ 17 Ω red). For de novo AF patients, wide antral circumferential ablation (WACA) of the PVs was performed and additional lesions sets, including posterior wall isolation, anterior mitral line, and superior vena cava isolation, were added at the discretion of the operator. For redo procedures, previous lesion sets were checked and reisolated where necessary. Additional lines were performed at the discretion of the operator. CTI ablation was performed depending on the patient’s history of atrial flutter or if atrial flutter spontaneously occurred during the procedure.

### Zero-fluoroscopy workflow

**Fig. 1:**
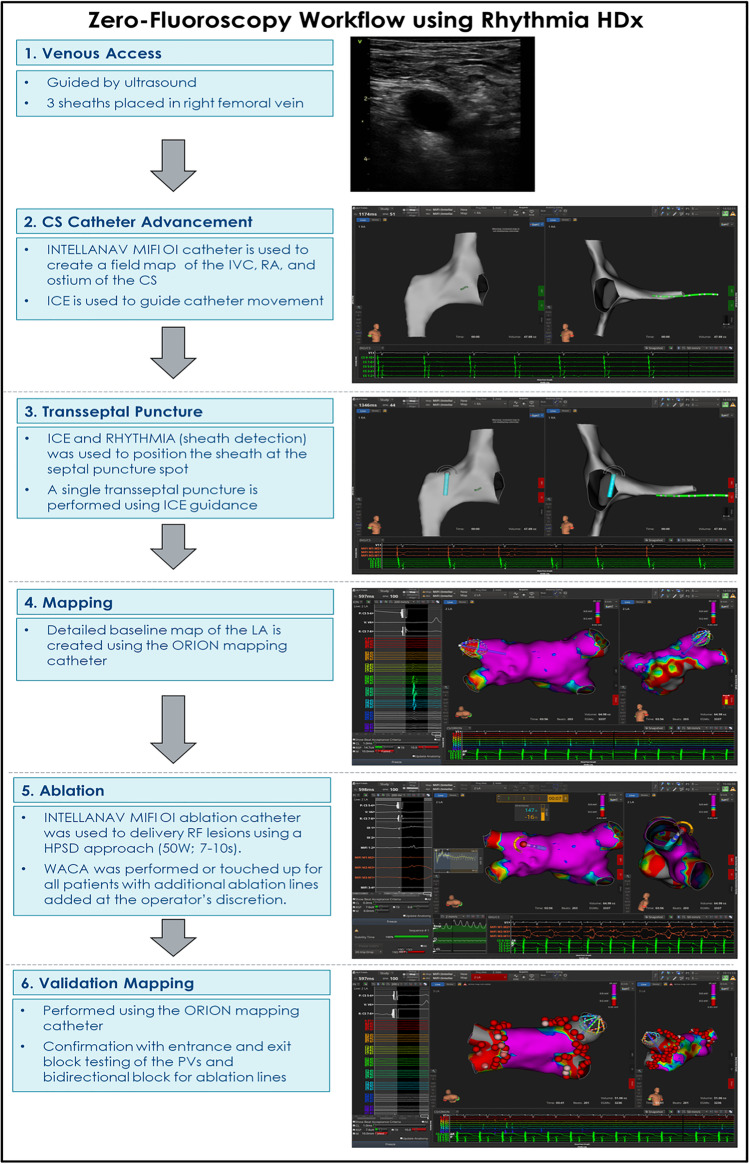
Zero-fluoroscopy workflow using the RHYTHMIA HDx mapping system

The workflow is described in the following steps: (1) venous access, (2) coronary sinus (CS) catheter placement, (3) transseptal puncture, (4) baseline mapping, (5) RF ablation, and (6) validation mapping (Fig. 1).

#### Venous access

Femoral venous access using an 18 g needle was guided by ultrasound. Three short access sheaths (6F, 8F, and 9F) were placed in the right femoral vein using the modified Seldinger technique. The steerable sheath was advanced to the pelvis prior to introducing the INTELLANAV MIFI OI catheter.

#### CS catheter placement

The INTELLANAV MIFI OI catheter was advanced from the femoral vein access to the inferior vena cava (IVC) and into the right atrium using magnetic tracking and visualized using the RHYTHMIA HDx mapping system, which can track and visualize catheter movement below the field magnet. The catheter was used to create anatomy and an impedance field map of the IVC, right atrium, and the ostium of the coronary sinus with ICE used as a guide from the right atrium. Using the impedance field map, the coronary sinus catheter was advanced and tracked on the RHYTHMIA mapping system into the right atrium, and then using RAO and LAO views on the mapping system, the catheter was placed in the coronary sinus for pacing and recording.

#### Transseptal puncture

**Fig. 2:**
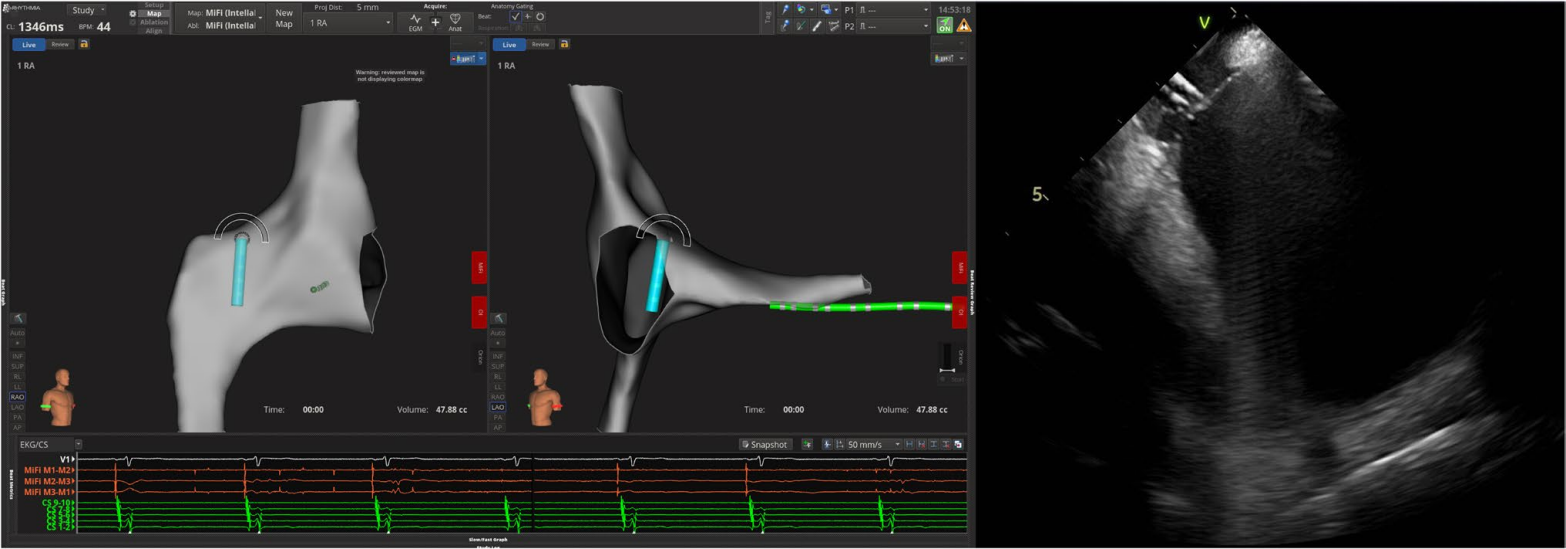
An example of the transseptal puncture guided by high density mapping (left) and ICE (right)

Prior to the transseptal puncture, a heparin bolus of 120 units/kg was given with repeat doses administered as necessary to achieve an activated clotting time greater than 350 s. The ablation catheter was advanced to the right atrium and placed against the septal wall using ICE and RHYTHMIA to guide placement (Fig. 2). Once positioned, the sheath was advanced over the tip of the catheter, using the RHYTHMIA sheath detection feature to guide localization. The sheath detection feature provides real-time feedback on electrode coverage by the sheath with a visual indicator on the mapping system. This feature informs the user when each of the catheter electrodes is within the sheath. Keeping the tip of the sheath at the puncture spot of the fossa ovalis, the ablation catheter was swapped out for the dilator loaded with the transeptal needle, looking for tenting of the septal wall on ICE. The physician operator maintained the positioning of the ICE catheter while manipulating the transseptal sheath. A single transseptal puncture is performed, and the sheath advanced into the left atrium under ICE visualization.

#### Baseline mapping

Prior to mapping, if presenting in AF, the patient was cardioverted to sinus rhythm. A detailed baseline map of the left atrium was created using the ORION mapping catheter, using ICE and RHYTHMIA to guide the localization of the veins and appendage, and entire left atrium. The baseline map was performed with coronary sinus catheter pacing at 600 ms for rapid data acquisition. If there was spontaneous recurrence of AF post cardioversion or during left atrial mapping, anatomy mapping was completed in AF.

#### RF ablation

**Fig. 3:**
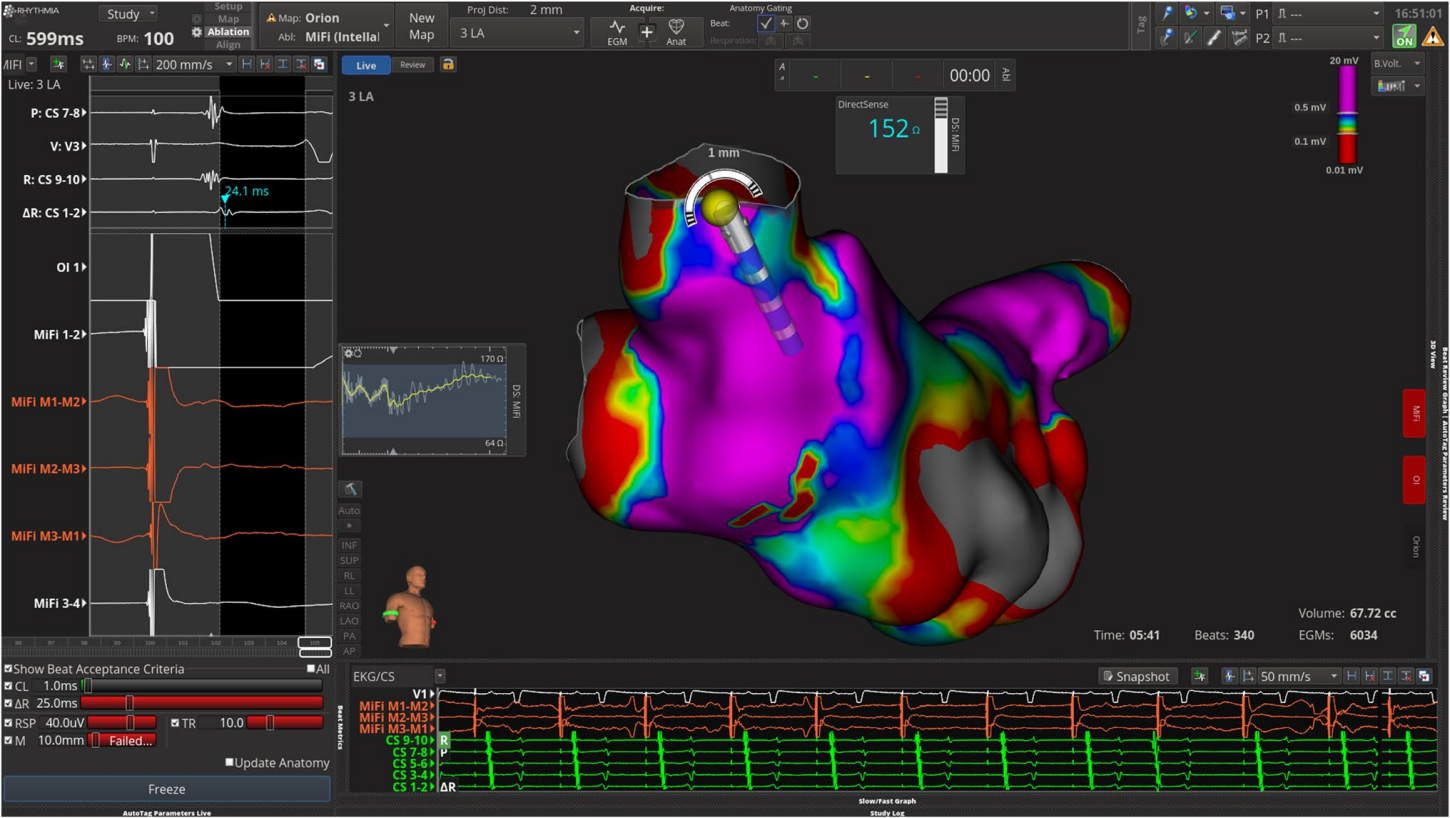
An example of phrenic nerve capture (yellow tag) identified using the RHYTHMIA mapping system based on locations of reduced tidal volumes

Prior to ablation, septal pacing was performed to identify and mark locations of phrenic nerve capture (yellow tag, Fig. 3) based on locations of reduced tidal volumes. Then, paralytics were administered to enable low tidal volume ventilation for better catheter stability while performing ablation. Baseline blood pool local impedance values were captured using the INTELLANAV MIFI OI ablation catheter before delivering RF lesions using a HPSD approach (50 W; 7–10 s). WACA was performed or touched up for all patients with additional ablation lines added at the operator’s discretion. Ablation tags were placed where the Autotag criteria were met (as previously described). The ablation lesions were guided by local impedance graphs to demonstrate acceptable variability along with local electrograms recorded on the mini electrodes and acceptable baseline local impedance targeting values 10–15% above blood pool impedance with variability less than 20 ohms. If the local impedance drop was less than 10 Ω, a contiguous ablation lesion with higher baseline impedance was targeted. Ablation lesions were stopped if the local impedance drop exceeded 25 Ω or if the local impedance drop did not reach 5 Ω after 5 s. The ablation catheter was used to perform simultaneous high output pacing (20 mA) on the posterior wall during ablation to evaluate loss of capture and EGM abolition. Pacing from the MiFi electrodes was performed to confirm exit block upon completion of WACA. Additional RF applications were delivered with simultaneous pacing and ablation of areas with residual tissue capture.

#### Validation mapping

Validation mapping was performed using the ORION mapping catheter. Across patients, activation mapping time averaged 3.0 ± 1.3 min. Confirmation of acute isolation was performed using entrance and exit block testing of the pulmonary veins and bidirectional block for ablation lines.

### Clinical outcomes

Procedural characteristics including procedure time, RF ablation time, and fluoroscopy time were recorded for each patient. Procedure time is defined as time from venous access to catheter removal from the heart. Procedure success was defined as achieving entrance and exit block of the pulmonary veins as confirmed with the ORION mapping catheter. In the event of posterior wall isolation, the mapping and ablation catheters were used together to confirm complete isolation. The ablation catheter was used to perform simultaneous high output pacing (20 mA) on the posterior wall during ablation to evaluate loss of capture and EGM abolition. In cases where CTI was performed, success was defined as bidirectional block with the ORION. Acute procedural complications and safety events through discharge (4–6 h post-procedure) were recorded.

### Statistics

Continuous variables are summarized as mean ± standard deviation. Statistically significant differences were identified using an unpaired *t*-test. Categorical variables are presented as proportions, and comparisons were performed using a *z*-test.

## Results

### Patient characteristics

Baseline patient characteristics for the heterogenous AF population (*n* = 72) treated in this cohort are shown in Table 1 by AF indication. Thirty-one cases (42%) were paroxysmal AF, and the remaining 41 (58%) were persistent AF, with no significant differences in baseline characteristics between groups. De novo AF ablation procedures were performed in 59 patients (82%).

**Table 1:** Patient characteristics

Characteristic	All (*N* = 72)	PAF (*N* = 31)	PersAF (*N* = 41)	*P*-value
Age (years)	67 ± 12	66 ± 11	67 ± 13	0.55
Gender, male [*N* (%)]	47 (66%)	19 (66%)	28 (66%)	0.54
Weight (kg)	100 ± 26 (*n* = 44)	98 ± 29 (*n* = 19)	101 ± 24 (*n* = 25)	0.72
Indication [*N* (%)]				
Paroxysmal AF	31 (42%)	31	-	─
Persistent AF	41 (58%)	-	41	─
Procedure type [*N* (%)]				
De novo	59 (82%)	25 (81%)	34 (83%)	0.80
Redo	13 (18%)	6 (19%)	7 (17%)	─
Comorbidities [*N* (%)]				
Diabetes mellitus	5 (7%)	3 (10%)	2 (5%)	0.43
Hypertension	34 (47%)	11 (36%)	23 (56%)	0.084
Stroke/TIA	2 (3%)	1 (3%)	1 (2%)	0.84
Structural heart disease	12 (17%)	4 (13%)	8 (20%)	0.45
Heart failure	13 (18%)	7 (23%)	6 (15%)	0.38

### Procedural characteristics

Procedural characteristics are shown in Table 2. The average procedure was 73.7 ± 16.2 min for all patients treated, with similar average procedure times for de novo and redo cases (73.6 ± 17.0 and 74.1 ± 12.5 min, respectively). Average procedure time was significantly shorter in paroxysmal AF patients compared to persistent AF patients (66.9 ± 13.5 vs 78.9 ± 16.3 min; *p* = 0.002). Paroxysmal AF patients also required significantly fewer RF applications (101 ± 29 vs 124 ± 34; *p* < 0.01) than persistent AF patients, which resulted in shorter RF times (16.0 ± 4.7 vs 19.4 ± 5.2 min; *p* < 0.01). All procedures were performed with zero fluoroscopy usage and no procedures required conversion to fluoroscopy.

**Table 2:** Procedural characteristics—paroxysmal AF and persistent AF patients

Procedural characteristics	All (*N* = 72)	Paroxysmal AF (*N* = 31)	Persistent AF (*N* = 41)	*P*-value
Procedure time (min)	73.3 ± 16.2	66.9 ± 13.5	78.9 ± 16.3	< 0.01
*CS placement to TSP (min)*	6.6 ± 8.1	5.6 ± 7.9	7.4 ± 8.2	0.35
*TSP to CS pull (min)*	67.1 ± 16.9	61.3 ± 14.3	71.5 ± 17.6	0.011
RF applications	114 ± 34 (*n* = 70)	101 ± 29 (*n* = 30)	124 ± 34 (*n* = 40)	< 0.01
Total RF time (min)	18.0 ± 5.2 (*n* = 70)	16.0 ± 4.7 (*n* = 30)	19.4 ± 5.2 (*n* = 40)	< 0.01
Average RF lesion time (s)	9.5 ± 0.8 (*n* = 70)	9.5 ± 0.8 (*n* = 30)	9.5 ± 0.8 (*n* = 40)	0.73
Validation mapping time (min)	2.9 ± 1.3 (*n* = 70)	2.7 ± 1.0 (*n* = 30)	3.1 ± 1.4 (*n* = 40)	0.25
Fluoroscopy time (min)	0	0	0	─
Effective dose (mSv)	0	0	0	─

*CS*, coronary sinus; *TSP*, transseptal puncture; *RF*, radiofrequency

### Acute outcomes

Catheter ablation was successfully performed in all 72 patients with a breakdown of ablation strategies and outcomes shown in Table 3. All de novo patients had their pulmonary veins isolated. For redo procedures, previous lesions were checked and reisolated where necessary. Additional ablations outside of the PVs were performed in 69% (50/72) of cases. All anatomical targets were successfully isolated using the HPSD, zero-fluoroscopy workflow. First pass isolation rates of the left and right PVs were 90% (56/62) and 91% (60/66), respectively. There were no significant procedure-related complications prior to discharge (Table 3).

**Table 3:** Acute efficacy and safety

Acute outcomes	
Efficacy – acute isolation	
Left pulmonary veins	62/62 (100%)
First pass isolation	56/62 (90%)
Right pulmonary veins	66/66 (100%)
First pass isolation	60/66 (91%)
Posterior wall	39/39 (100%)
CTI line	19/19 (100%)
Safety	
Death	0 (0%)
Cerebral vascular event	0 (0%)
Cardiac tamponade	0 (0%)
Pericarditis	0 (0%)
Hematoma	0 (0%)
Deep vein thrombosis/pulmonary embolism	0 (0%)

## Discussion

A zero-fluoroscopy workflow using the RHYTHMIA mapping system was shown to be feasible and demonstrated acute safety for ablation treatment in this heterogenous AF population. All patients were successfully treated with first pass isolation rates of 90% and 91% for the left and right PVs, respectively. No patients required conversion to fluoroscopy, and, importantly, there were no acute complications.

In this experience, all cases were performed without contact force sensing since it is not yet commercially available with this mapping system in the USA. Despite not having contact force guidance, we demonstrated an excellent safety profile with no acute complications through the use of HDM, ICE, and local impedance monitoring. Local impedance provided feedback to guide RF energy delivery to achieve effective lesion formation and maintain patient safety. Ultimately, this was a very efficient workflow for PVI, and once available, a catheter combining contact force and local impedance will supplement this zero-fluoroscopy workflow, especially for operators who have trained with contact force guidance.

Our experience here is the first to describe a zero-fluoroscopy workflow for AF ablation with the RHYTHMIA mapping system. Although there was no comparative group using conventional fluoroscopy guidance, the procedure times shown here were aligned with previously published results [11, 14, 18, 19]. One study compared 80 paroxysmal AF patients between two workflows and found procedure times were similar (mean procedure time: 92.5 min for zero-fluoroscopy vs 99.9 min for fluoroscopy-guided) [11]. In a recent study by Lui et al., 200 AF ablation procedures were performed with zero fluoroscopy with a mean procedure time of 106.2 min [19]. This study included a heterogenous patient population with 82% of patient requiring ablation outside of the pulmonary veins [19]. This study included comparison to a control group of 50 AF patients treated with fluoroscopy-guided ablation; interestingly, these patients had a significantly longer average procedure time of 127.9 min than those under zero fluoroscopy [19].

In this experience, there was no significant prolongation of procedure times as a result of eliminating radiation exposure. In fact, procedure times reported here were under those seen in previous studies [11, 14, 19] with a mean duration of 73.7 min (range: 40.3 to 126.6 min). Furthermore, this was a diverse AF patient population which included 69% (50/72) of cases requiring the ablation of additional non-pulmonary vein triggers (118 ± 37 applications) and 58% (41/72) of persistent AF cases (124 ± 34 RF applications). It is important to note that procedure times may be longer initially during the learning curve [9]. However, these experiences demonstrate that eliminating radiation does not prolong procedure times.

Despite numerous studies demonstrating the feasibility and safety of zero-fluoroscopy for cardiac ablation [11–14, 18–20], fluoroscopy is still often used in AF ablation procedures for manipulating the catheter, transseptal puncture, and visualization of the left atrium [9]. However, with the number of AF ablation procedures on the rise and patients often requiring multiple ablation procedures in their lifetime to treat recurrence of different cardiac arrhythmias, the hazards of radiation should not be overlooked.

Radiation exposure for both physicians and patients involves risk of biological damage. It is well-known that there are both deterministic and stochastic effects of radiation exposure. Deterministic effects include radiation skin injury, cataracts, and hair loss which occur once a radiation threshold is exceeded. Stochastic effects of radiation, such as cancer, are not associated with a specific radiation threshold but instead the risk increases proportionally with the exposure [20]. For instance, the absolute lifetime risk of fatal cancer for an adult increased by 0.05% for every 10 mSv of exposure, and the average dose for AF ablation is 15 mSv [21]. Guidelines encourage physicians to minimize the patient’s radiation exposure and subsequent risk of radiation injury by following the ALARA principle. The best practice that minimizes this risk follows three basic principles: (1) there is no known absolutely safe dose of ionizing radiation; (2) the smaller the dose, the less the risk of an adverse effect; and (3) incremental radiation exposures have cumulative effects [22].

The field of cardiac electrophysiology is moving towards a reduction of radiation used in procedures while also exploring novel energy sources such as pulsed field ablation. It is critical to achieve a solid foundation on simplification of the procedure and proficient use of other imaging modalities outside of fluoroscopy. Furthermore, components of the workflow described here, specifically the CS placement, transseptal puncture, and left atrial catheter maneuvering, are independent of energy source and can help eliminate the reliance of radiation.

### Limitations

The focus of this experience was to detail a zero-fluoroscopy workflow using the RHYTHMIA mapping system combined with ICE. Although initial data shows this technique to be acutely safe and effective in this clinical experience, there are several limitations. This was a retrospective description of patients from a single center and single operator. Further studies are needed to evaluate the feasibility and safety of this workflow by experienced operators at multiple centers. Lastly, follow-up for this experience was limited to patient discharge, which was 4–6 h following the procedure, and there are no long-term clinical outcomes reported as part of this experience.

## Conclusion

Our workflow combining ICE and EAM using the RHYTHMIA mapping system enables the use of zero-fluoroscopy for AF ablation. All procedures successfully achieved acute electrical isolation with no acute safety events reported and with reasonable procedure durations. This approach for AF ablation can eliminate the radiation exposure to patients and medical staff and reduce the physical burden associated with wearing protective lead aprons. Further study is needed to evaluate the long-term clinical outcomes.
